# Secondary sclerosing cholangitis after COVID-19 pneumonia: a report of two cases and review of the literature

**DOI:** 10.1007/s12328-022-01687-5

**Published:** 2022-08-11

**Authors:** Ulrike Bauer, Desislava Pavlova, Rami Abbassi, Tobias Lahmer, Fabian Geisler, Roland M. Schmid, Ursula Ehmer

**Affiliations:** Internal Medicine II, Klinikum rechts der Isar, Technische Universität München, Munich, Germany

**Keywords:** Cholangiopathy, COVID-19, ERCP, Liver transplantation, SC-CIP

## Abstract

Secondary sclerosing cholangitis in critically ill patients (SC-CIP) is a rare disease characterized by chronic cholestasis. The underlying pathophysiology of SC-CIP is not fully understood, and prognosis in severe cases remains poor with liver transplantation remaining the only curative treatment option. There is a growing amount of literature describing patients with chronic cholangiopathy after COVID-19 infection. The vast majority of the patients described in these reports were male and had a poor outcome. While the exact percentage of patients with COVID-19-related SC-CIP cannot be estimated accurately due to a lack of larger studies, an increase in patients with long-term complications of chronic cholestatic liver disease after severe COVID19-pneumonia can be expected in the upcoming years. Treatment options remain limited and further research is needed to improve the dismal prognosis of SC-CIP. Here, we present the cases of two patients who developed SC-CIP after prolonged intensive care unit stay due to severe COVID-19 pneumonia. Both patients required invasive ventilation for 31 and 141 days, respectively, as well as extra-corporal membrane oxygenation for 23 and 87 days. The patients suffered from jaundice and severe pruritus, and typical features of SC-CIP were present by MRCP and ERC. Repeated removal of biliary casts resulted in some alleviation of their clinical symptoms, but cholestasis parameters remain elevated. Furthermore, an increased liver stiffness was indicative of advanced fibrosis in both patients. In addition to these two case reports, we provide a concise review of the literature of SC-CIP after COVID-19 infection and discuss risk factors, treatment options and prognosis.

## Introduction

Secondary sclerosing cholangitis in critically ill patients (SC-CIP) is a rare disease with a poor prognosis. First described in 1997 [1], the pathophysiology of SC-CIP is still not fully understood and efficient therapeutic options are virtually non-existent. Ischemic injury, viral infections, and immunological changes all contribute to damage to the biliary epithelium, resulting in impaired bile flow, consecutive liver damage with the development of fibrosis and cirrhosis [2]. The most prominent clinical features in SC-CIP are pruritus and recurrent cholangitis as well as profound jaundice in more severe cases. Diagnosis is usually confirmed by elevated cholestasis parameters and magnetic resonance imaging cholangiopancreatography (MRCP). Characteristic diagnostic features are rarefication and dilatation of intrahepatic bile ducts with diffuse strictures—similar to the changes observed in primary sclerosing cholangitis [3]. To date, treatment is limited to the management of complications such as cholangitis or pruritus. In patients who develop cirrhosis and end-stage liver disease, transplantation remains the only therapeutic option [4, 5]. Mortality without transplantation is high with survival times between 17 and 40 months [6]. While previously an incidence of SC-CIP of 1:2000 has been reported in overall intensive care unit (ICU) patients [7] the rise in numbers of critically ill patients during the pandemic caused by Severe Acute Respiratory Syndrome Coronavirus 2 (SARS-CoV-2) has already translated into an increase in reports of SC-CIP cases.

In patients with coronavirus disease-2019 (COVID-19) who require hospitalization, a mild elevation of liver enzymes (< 2 × ULN) is frequently observed in up to 21–63% of cases [8–10]. However, a severe increase in transaminases and cholestasis parameter or impaired liver function are rare and almost always occur in patients who suffered from multi-organ failure.

## Case reports

**Fig. 1:**
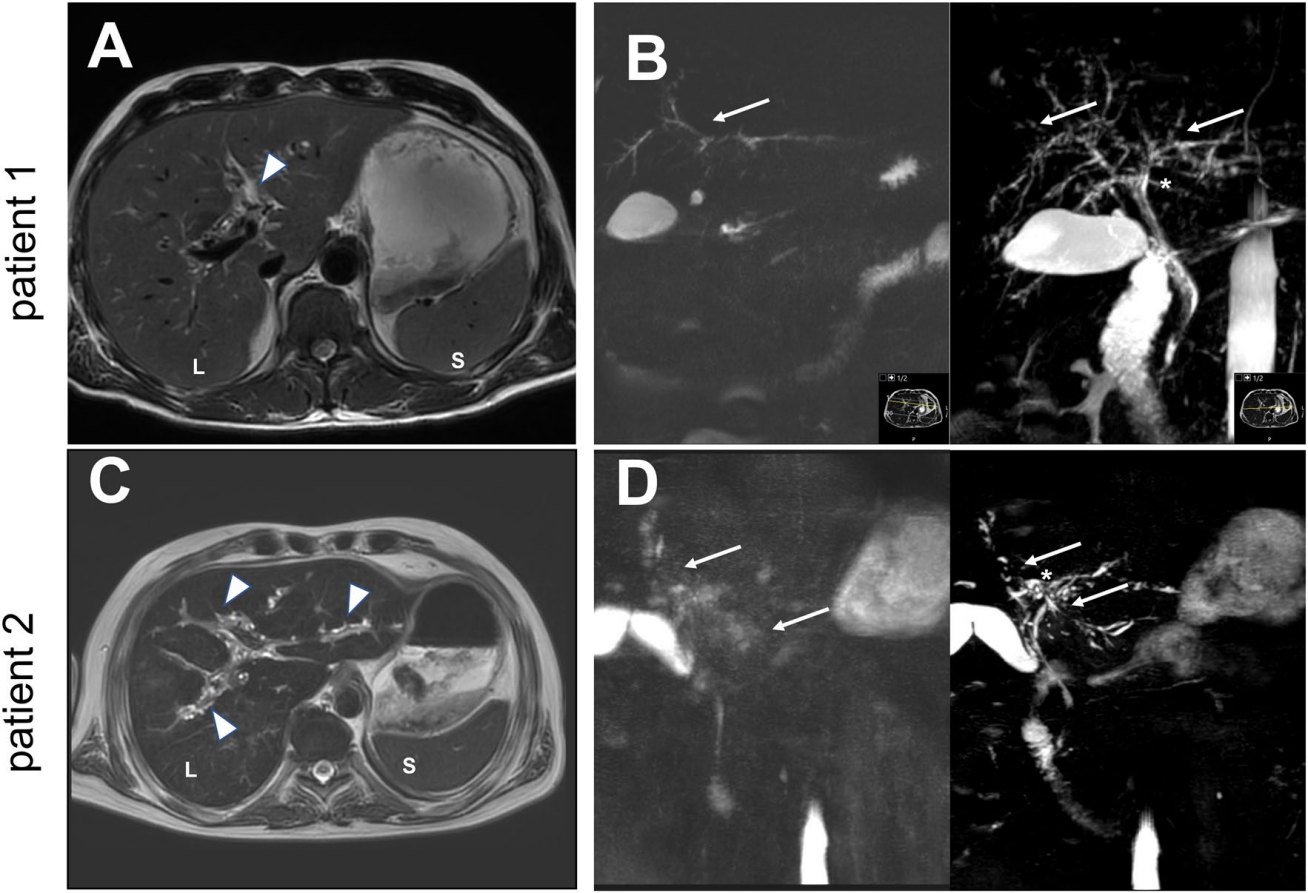
MRI **A** and **C** and MRCP **B** and **D** scan of patient 1 (top) and patient 2 (bottom), showing structural abnormalities of the bile ducts, such as intrahepatic multifocal strictures (arrows), bile duct ectasia (*), ductal wall thickening (arrow heads), irregular beading, and cast formation in the proximal common bile duct. *L* liver, *S* spleen

**Fig. 2:**
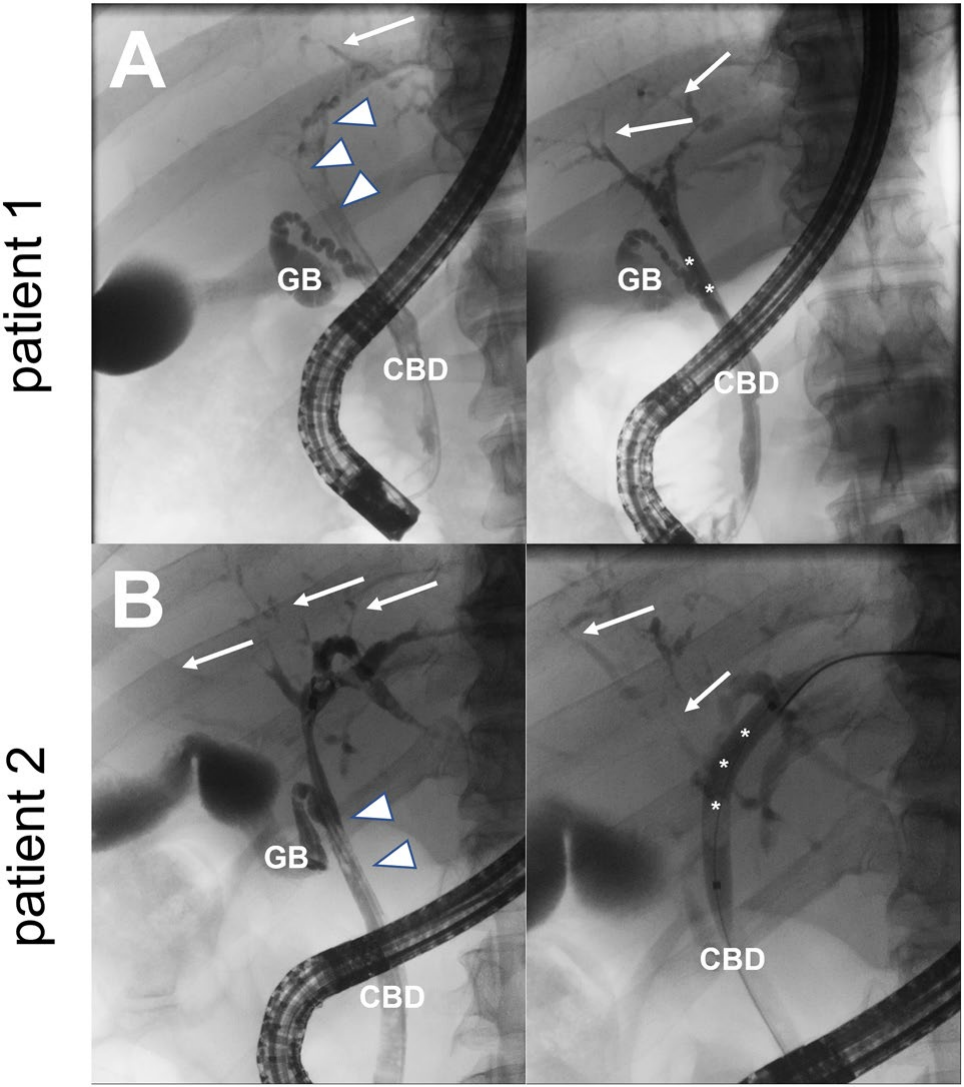
ERC of patient 1 **A** top and patient 2 **B** bottom 375 and 542 days, respectively, after initial hospital admission. Arrows show rarefication of bile ducts with arrowheads indicating bile duct casts, asterisks show balloon dilatation. *CBD* common bile duct, *GB* gall bladder

The first patient is a 48-year-old male with no known medical preconditions who presented to our hospital with COVID-19 pneumonia and mildly elevated liver enzymes (< 2 × ULN). His respiratory situation deteriorated rapidly, invasive ventilation was initiated a few hours after admission and needed to be continued for 31 days, complemented by extra-corporal membrane oxygenation (ECMO) for 23 days. During this time, liver enzymes increased considerably (maximum gGT 1,617 U/L, ALP 1,100 U/L, ALT 390 U/L, AST 320 U/L, bilirubin 1.4 mg/dL) and decreased slowly during recovery. The patient was discharged 75 days after initial admission with a persistent elevation of ALP and gGT as well as transaminases, but normal bilirubin levels (gGT 1050 U/L, ALP 1098 U/L, ALT 208 U/L, AST 88 U/L). More than ten months after ICU admission, the patient presented with mild jaundice, pruritus, and elevated cholestasis parameters (ALP 812 U/L, gGT 413 U/L, bilirubin 5.0 mg/dL) and transaminases (ALT 137 U/L, AST 118 U/L), but normal albumin and INR. MRCP showed profound structural bile duct abnormalities with multifocal strictures and dilated intrahepatic ducts (Fig. 1A and B). During ERC a huge bile duct cast lining the entire common hepatic bile duct (Fig. 2A) was removed. By imaging, there were no obvious signs of cirrhosis, but elastography (Fibroscan) revealed an enhanced liver stiffness, that could be due to advanced fibrosis (31.5 kPa, IQR 1.5 kPa), but might also be due to the presence of severe cholestasis.

The second patient is a 60-year-old male who was slightly overweight but did not suffer from any other medical preconditions. He developed severe COVID-19 pneumonia and required invasive ventilation for 141 days and ECMO for 87 days before he could be discharged 151 days after initial admission. The patient developed cholangitis complicated by multiple small liver abscesses that were treated with antibiotics and antimycotics. MRI and MRCP showed classical features of SC-CIP (Fig. 1C and D). ERC with the extraction of biliary casts was repeatedly performed—most recently 542 days after hospital admission—and showed rarefication of intrahepatic bile ducts (Fig. 2B). While bilirubin levels decreased after endoscopic treatment, the patient still presents with a reduced general condition and suffers from ubiquitous pruritus, which correlates with increased cholestasis parameters (ALP 760 U/L, gGT 720 U/L, bilirubin 4.0 mg/dL). Transaminases were only mildly elevated (ALT 80 U/L, AST 80 U/L) and transient elastography showed an increased liver stiffness (29.7 kPa, IQR 2.5 kPa) (Table 1). Both patients were prescribed ursodeoxycholic acid (UDCA). No liver biopsy was performed as diagnosis of SC-CIP was evident from ERC and MRCP.

**Table 1:** Overview of patient characteristics of two male patients with SC-CIP after severe COVID-19 pneumonia requiring ECMO treatment

	Patient 1	Patient 2
Age (years)	48	60
Sex	Male	Male
BMI (kg/m^2^)	35.91	27.76
Weight loss in kg (percent body weight)	43 (41%)	35 (41%)
Comorbidities	History of anabolic steroids	None
Preexisting liver disease	Mild impairment of liver enzymes (< 2 × ULN)	None
Invasive ventilation (days)	31	141
ECMO (days)	23	87
ERC	Removal of a huge bile duct cast (12 cm), picture consistent with SC-CIP	5 × removal of bile duct casts, picture consistent with SC-CIP
Cholangitis	None	One episode with development of liver abscess
Elastography	31,5 kPa, IQR 1,5 kPa F4	29,7 kPa, IQR 2,5 kPa F4

## Discussion

More than two years after the emergence of SARS-CoV-2, nine reports of individual cases and four case series describe patients who developed SC-CIP after COVID-19 infection. As summarized in Table 2, most of the patients were male (26/29, 90%) and the mean patient age was 55 years (SD ± 8 years). The majority of patients suffered from impaired liver function, with three patients requiring liver transplantation, and one being evaluated for transplantation.

**Table 2:** Summary of reports on SC-CIP after COVID-19 infection

Case reports					
Sex	Age (years)	BMI (kg/m^2^)	Follow-up (months)	Outcome	Ref
Male	47	51	11	Liver transplantation	[25]
Male	47	24	5	Liver transplantation	[26]
Male	64	30	17	Liver transplantation	[27]
Male	38	n/a	n/a	Evaluation for liver transplantation	[28]
Male	57	n/a	n/a	Cholangitis after hospital discharge	[29]
Male	59	n/a	6	Several ERC procedures with balloon trawl to remove sludge	[30]
Male	62	n/a	n/a	Several ERC procedures due to cholestasis	[31]
Female	54	n/a	5	Ketamine related SC-CIP	[15]
Female	61	n/a	4	Patient died	[32]

Case series					
Sex (m/f)	Mean age (years)	Mean BMI (kg/m^2^)	Median follow-up (months)	Outcome	Ref
3/1	61	25.35	4.7	2 patients died	[11]
4/0	n/a (48 -68 years)	n/a	n/a	1 patient died, liver transplantation in 2 patients	[12]
11/1	n/a	n/a	n/a	Liver transplantation in 1 patient	[13]
n/a	Median 59	Median 28	n/a	2 patients died, 2 patients suffered from biliary sepsis	[14]

*LT* liver transplantation

The first case series reports a high incidence of SC-CIP after severe COVID-19 pneumoniae. In this cohort, four out of 34 patients developed SC-CIP with ischemic bile duct damage as assessed by imaging and histopathology. After a median follow-up of 10 months after initial diagnosis, two of the four patients had died [11]. The second report evaluated a larger cohort of 114 COVID-19 patients admitted to ICU, of which four patients developed SC-CIP. All patients were male and two of them required liver transplantation. One of the remaining two patients had died by the time of manuscript submission. Diagnosis was confirmed by histopathology and the patient showed typical hallmarks of SC-CIP by imaging [12]. In a third case series of 12 patients who developed bile duct damage after severe COVID-19 pneumonia, radiographic and histological findings were consistent with large duct obstruction but without clear loss of intrahepatic bile ducts. Liver biopsy was performed on four patients to confirm the diagnosis. Five patients suffered from more severe complications of SC-CIP such as hepatic insufficiency or recurrent bacterial cholangitis; one successfully underwent liver transplantation [13].

Interestingly, a case series of five patients differs from other reports of COVID-19-associated SC-CIP—suggesting that ketamine exposure is a critical contributor to the development of cholangiopathy with SC-CIP-like features. Of the five patients suffering from COVID-19-associated SC-CIP all had received ketamine with a mean exposure of 16 days (range 6–26 days). In the following, two of the patients died, two developed biliary sepsis, and one patient suffered from pruritus. Of note, liver damage seemed to rise with higher ketamine doses [14]. A similar phenotype was described in a 54-year-old patient with COVID-19 who developed SC-CIP after ketamine exposure [15]. Importantly, ketamine-induced cholangiopathy has been described before the emergence of SARS-CoV2. After seven years of ketamine abuse, a 32-year-old female without preexisting liver disease developed intrahepatic bile duct strictures [16] and a 21-year-old male began suffering from cholangiopathy after nine months of alcohol and ketamine abuse [17]. Interestingly, histopathological changes such as thickened membranes of the interlobular bile ducts were consistent with SC-CIP, but no bile duct strictures were observed by MRCP [17]. The pathophysiology of ketamine-induced cholangiopathy remains unclear. Preclinical studies hint at increasing the frequency of sphincter of oddi contractions upon ketamine exposure [18]. In humans, long-term ketamine abuse seems to be required to induce bile duct changes in otherwise healthy individuals. Even with ketamine being a widely used anesthetic, reports of bile duct damage after short-term use are virtually non-existent from the pre-COVID-19 era. Nevertheless, even a low level of bile duct toxicity could be potentiated in the presence of other conditions that predispose to SC-CIP such as hypoxemia or septic cholangiopathy. Especially in patients with severe COVID-19 pneumonia and rising liver enzymes, ketamine toxicity should be considered.

The picture is complicated by reports of a disease phenotype consistent with sclerosing cholangiopathy by clinical appearance but lacking the classical features of SC-CIP by imaging. In a case report of a 29-year-old female who suffered from severe COVID-19 pneumonia, the presence of cholangiopathy without fibrosis was confirmed by liver biopsy, but no biliary obstruction was evident by MRCP [19]. In three patients with COVID-19-associated cholangiopathy (2 male, 1 female; 25–40 years) cholangiocyte injury and fibrosis were observed by histopathology. However, none of the biopsies showed neutrophilic inflammation or portal edema [20], a characteristic feature regularly observed in patients with acute bile duct injury [21, 22]. Due to this unique histopathological pattern, the authors suggest a new disease entity in these patients [20].

While the case series described above and several case reports suggest a high rate of SC-CIP of up to 3.5% of ICU patients with COVID-19 [12], large-scale data remains limited. At our university hospital, 168 of 269 patients admitted to ICU between 17 March 2020 and 5 November 2021 survived (62%). 16 ICU patients were treated with ECMO of whom only three survived (19%) and one of the surviving patients developed SC-CIP. However, the two others had no elevation of ALP after discharge from ICU (75 U/l, respectively, 72 U/l). Of the overall patient cohort, ALP levels at admission and discharge from the hospital were available from 105 patients. Mean ALP was 89 U/L (range 30–260 U/L) at hospital admission and 162 U/l (range 41 U/L–2095 U/L) at discharge. While the majority (76%) had a normal ALP at discharge, 16% showed slightly increased ALP levels (< 2 × ULN; 17/105), and 5% a severe increase: 2–5 × ULN (5/105) and 3% an ALP > 5 × ULN (3/105). In contrast, at the time of admission, 87% had a normal ALP (91/105) and no ALP above 2 × ULN was observed. Though follow-up information was not always for all cases, these data suggest a high rate of bile duct damage (defined by an ALP > 2 × UNL) in about 8% of patients.

SC-CIP remains a disease that is poorly understood. As evident from the heterogeneous clinical picture described in various case reports there are probably multiple contributors to bile duct damage in COVID-19 patients. Several variables such as the duration of ischemic injury, the severity of inflammation, secondary infectious complications of viral pneumonia, the use of drugs such as ketamine, sex, age, and possibly genetic factors might all influence the severity and even clinical and histological features of bile duct damage—resulting in a heterogenous appearance of COVID-19-associated cholangiopathy. While the clinical symptoms in reported cases seems to be are similar, imaging by MRCP or MRI seems to be variable and does not always show biliary strictures [19]. Likewise, histological features seem to differ from those of classical sclerosing cholangitis in some cases. Given the limited number of reports, it remains unclear if these findings translate into a novel disease entity or simply represent different characteristics in the heterogenous spectrum of SC-CIP.

In the following of the COVID-19 pandemic, it is highly likely that cases of this formerly rare disease will increase. Even though the number of cases with a poor prognosis might be overestimated due to the high number of reports of severe SC-CIP cases, elevation of liver enzymes was common at our center after prolonged invasive ventilation for COVID-19. Therefore, the presence of an underlying SC-CIP should be investigated in patients that retain elevated cholestasis parameters—especially since the diagnosis might have been initially overlooked due to multiple co-morbidities at the time of discharge. Even with few treatment options, monitoring these patients for the development of chronic liver damage could help in the diagnosis and management of long-term complications.

To date, treatment options in SC-CIP remain limited. Most of the patients described in the literature received UDCA, but it remains unclear whether UDCA improves liver function. Similar to the treatment in patients with primary biliary cholangitis, UDCA might protect cholangiocytes from inflammation [23]. A combination of UDCA and ERC might improve prognosis, though it cannot stop disease progression [24]. However, the benefit of ERC in SC-CIP still remains unclear though it can improve biliary obstruction in the presence of biliary casts [4]. Possible ERCP complications such as pancreatitis or an increased risk of ascending cholangitis after papillotomy must be weighed against the benefits. With the current lack of efficient treatment options, the prognosis of severe SC-CIP remains grim with liver transplantation being the only curative option. In less severe cases, the prognosis might be limited due to long-term secondary complications such as recurrent episodes of cholangitis or fibrotic remodeling of the liver leading to the development of cirrhosis and associated complications. If and how the outcome in these patients can be improved remains unknown to date.
